# Ophthalmodynamometry for ICP prediction and pilot test on Mt. Everest

**DOI:** 10.1186/1471-2377-10-106

**Published:** 2010-11-01

**Authors:** Henry W Querfurth, Philip Lieberman, Steve Arms, Steve Mundell, Michael Bennett, Craig van Horne

**Affiliations:** 1 Dept of Neurology, Rhode Island Hospital, Warren Alpert Medical School, Brown University Providence, RI 02903 USA; 2 Dept of Cognitive and Linguistic Sciences, Brown University, Providence, RI 02912 USA; 3 MicroStrain Inc. Williston, VT 05495 USA; 4 Dept of Neurosurgery, Caritas St. Elizabeth's Medical Center, Tufts University School of Medicine. Boston MA 02135 USA

## Abstract

**Background:**

A recent development in non-invasive techniques to predict intracranial pressure (ICP) termed venous ophthalmodynamometry (vODM) has made measurements in absolute units possible. However, there has been little progress to show utility in the clinic or field. One important application would be to predict changes in actual ICP during adaptive responses to physiologic stress such as hypoxia. A causal relationship between raised intracranial pressure and acute mountain sickness (AMS) is suspected. Several MRI studies report that modest physiologic increases in cerebral volume, from swelling, normally accompany subacute ascent to simulated high altitudes.

**Objectives:**

1) Validate and calibrate an advanced, portable vODM instrument on intensive patients with raised intracranial pressure and 2) make pilot, non-invasive ICP estimations of normal subjects at increasing altitudes.

**Methods:**

The vODM was calibrated against actual ICP in 12 neurosurgical patients, most affected with acute hydrocephalus and monitored using ventriculostomy/pressure transducers. The operator was blinded to the transducer read-out. A clinical field test was then conducted on a variable data set of 42 volunteer trekkers and climbers scaling Mt. Everest, Nepal. Mean ICPs were estimated at several altitudes on the ascent both across and within subjects.

**Results:**

Portable vODM measurements increased directly and linearly with ICP resulting in good predictability (r = 0.85). We also found that estimated ICP increases normally with altitude (10 ± 3 mm Hg; sea level to 20 ± 2 mm Hg; 6553 m) and that AMS symptoms did not correlate with raised ICP.

**Conclusion:**

vODM technology has potential to reliably estimate absolute ICP and is portable. Physiologic increases in ICP and mild-mod AMS are separate responses to high altitude, possibly reflecting swelling and vasoactive instability, respectively.

## Background

Several methods for non-invasive measurement of ICP have been described but none have come to clinical use. They include i) tympanic membrane displacement (TMD) [1], ii) ultrasonic detection of skull pulsations [2], iii) transcranial Doppler (TCD) ultrasonography of the middle cerebral artery (MCA) [3-9], iv) visualization of retinal venous pulsations [10], v) optic nerve sheath diameter (ONSD) measurement by ultrasound [11], vi) cochlear-microphonics [12], and vii) blood flow pulsations [13]. Small signal size and baseline variability among healthy individuals, due in part to anatomic variances affecting CSF communication between compartments, have limited the application of some of these methods (e.g. TCD [14,15]; TMD [1]: and ONSD [16]. Moreover, they cannot provide absolute ICP prediction.

Central retinal vein (CRV) pressure measurements quantifiably predict *actual *ICPs within a physiologic range [17,18]. Increases in ICP directly affect the collapsible and valveless venous system draining the orbit into the cavernous sinus. As brain pressure rises, so does the resistance to retinal venous outflow. CRV pressure rises proportionately to just overcome this ICP effect on outflow resistance. Venous ophthalmodynamometry (vODM) is a method of manually raising intraocular tension (IOT) above the resting intraocular pressure (IOP), to a point at which the central retinal vein is observed to collapse. The pressure value at the point of collapse is termed the venous outflow or venous occlusion pressure (VOP) and is found to linearly predict absolute ICP. The technique involves applying slight graded pressure to the orbital sclera using a calibrated force transducer that relies on either a compression-based spring gauge (see below) or vacuum cup technology to manipulate intraocular tension. Standard ocular tonometry and ophthalmoscopy are additionally employed to derive resting IOP and visualize the instantaneous moment of vein collapse, respectively. We reported the calibration results of a prototype venous ophthalmodynamometer in a unblinded pilot study of 6 ICU patients [18]. vODM was found to predict absolute intracranial pressure with a correlation coefficient ~0.9. Nevertheless, the clinical utility of any of these methodologies to predict actual ICPs awaits the demonstration of a proportionate change to a continuous physiologic variable.

Shared clinical symptoms and circumstantial evidence suggest a causal relationship between raised intracranial pressure (ICP) and acute mountain sickness (AMS). The main symptom, headache, as well as nausea, anorexia, fatigue and dizziness also overlap with migraine. The pathophysiology of AMS is unknown, however intracranial hypertension from cerebral swelling and hypoxia has been suspected to be an important factor [19,20]. The mechanisms behind the underlying cerebral swelling and variable vasogenic edema are postulated to be cerebral vasodilation, increased blood flow (CBF) and cerebral blood volume in combination with hydrostatic pressure- and oxyradical or cytokine-induced blood brain barrier (BBB) permeabilization [21-23]. However, no studies to date have provided absolute ICP data as a function of altitude and correlated this with AMS severity in the field. The extreme form of AMS, high altitude cerebral edema (HACE), is associated with increased ICP. Increases in cerebrospinal fluid (CSF) pressure from 6 to 21 cm H_2_0 above baseline were recorded in one classical study [24]. Vasogenic edema in white matter structures, particularly the splenium of the corpus callosum, were noted on MRI in another series of HACE cases [25]. Whether the latter is causal of symptoms and intracranial hypertension or a correlated phenomenon is not clear.

Although most MRI studies do not find signs of raised ICP or generalized edema in normal subjects at simulated altitude, changes occur to suggest mild global brain swelling. MRI studies in non-exercised subjects exposed to a simulated altitude of 4500 m (either normobaric hypoxia; FIO2 = 12% or hypobaric hypoxia; 429 mm Hg) for 16-32 hours show consistent increases in brain volume, either global or grey matter specific, across all subjects ranging from 0.6 - 2.7% [23,26,27] and 1.1 - 5.8% [28]. This swelling arises from increased cerebral blood volume (~7 cc) and/or parenchymal water content (vasogenic edema) [27]. Some [22,29], but not all studies [30], confirm the latter. Nevertheless, in agreement with general swelling a compensatory 10% reduction in CSF volume is observed in all subjects [30]. Taken together, these findings suggest that physiologic swelling under hypoxic conditions may not be sufficient to account for mild to moderate AMS symptoms. In contrast, there is much less data on ICP changes with altitude owing to the invasiveness of obtaining direct numbers. Lumbar CSF pressures remained unchanged or were inconclusive compared to normoxic values in individuals at simulated 4500 m, with or without mild -mod AMS [19,26,31]. In a single case with an implanted telemetric ICP monitor at 5030 m, ICP increased to upper normal levels at rest and further still with exertion [32]. The question whether a physiologic rise in absolute ICP accompanies the increase in cerebral volume and if this correlates with AMS symptoms can be addressed with vODM.

The dual primary aims of this study were to validate and calibrate a novel portable venous ophthalmodynamometer and then use it to non-invasively estimate ICP values in subjects at various altitudes, a test of the physiologic response to hypobaric hypoxemia. Our previously reported venous ophthalmodynamometer was modified and re-calibrated against a new set of neurosurgical patients, most with hydrocephalus, in the hospital setting. Actual or 'gold standard' ICP values were recorded using conventional invasive catheters and continuous monitoring or from spinal tap at the time of the non-invasive test. Additional validation involved training several operators to collect the vODM data while 'blinded' to the actual ICP. We next conducted a clinical field test on volunteer trekkers and climbers on the approach to and at Camp 2, Mt. Everest, Nepal in 2007. A secondary aim examined the relationship of ICP to symptoms of AMS. We found that by vODM, ICP increases physiologically with ascent.

## Methods

### Venous ophthalmodynamometry

#### vODM device

In compression-based vODM [18], force is transduced through a miniature spring-loaded Differential Variable Reluctance Transducer (DVRT^®^) (MicroStrain, Inc. Williston, VT). Briefly, the spring provides a precise counter force to the linear displacement of a plunger core within a set of energized coils. This sensor unit has a force resolution of approximately 0.5 milliNewton and is housed within a smaller than pencil-sized, finger-held probe. A signal-conditioning card converts the linear displacement into an output signal calibrated in grams that is accurate to 1/10 gram. This sensitivity makes it possible to record within the low range of pressures applied to the orbit (sclera) necessary to produce the visible collapse of retinal venous outflow. The probe tip that contacts the sclera is from polished stainless steel (a convex, 6.3 mm dia. footplate) and is threaded for easy removal and sterilization or disinfection in chlorohexidine.

The prototype vODM was improved upon by: a) powering it with two rechargeable Lithium polymer (14.8 V, 2150 mAh) batteries. The electrical isolation also made it convenient for patient contact in the ICU and full portability into the field, b) designing a finger activated button-type switch to freeze the display readout at the moment venous occlusion is reached and then to reset it. This replaces the original foot petal switch, making it possible to measure reclined subjects at ground level. It was also found to improve the intra-operator reliability of VOP measurements due to the finer eye-hand control, c) enclosure within an IP67 environmentally sealed box (Additional file 1). It was pretested over a temperature range of -4°C to 50°C by calibrating the output voltage against the force (gms) applied to an analytical balance. The relationship was completely linear (1:1) with minimal drift (results not shown).

#### Technique

The eye is first anesthetized with topical proparacaine HCL (0.5%) and then dilated with Tropicamide (Mydriacyl, 1%), but neither is an absolute requirement. A single resting intraocular pressure reading was obtained in all subjects using a TonoPen tonometer (Reichert Co.) before the ODM measurement. A Welsh-Allyn portable ophthalmoscope was temporarily attached to the finger activated freeze display switch. Next, the intraocular tension (IOT) is incrementally elevated by graded application of the ODM plunger to the lateral orbit (sclera) until one of the retinal veins was observed to suddenly collapse through the hand-held direct ophthalmoscope. The instantaneous reading or venous occlusion pressure (VOP, load in grams) is frozen using the finger-activated switch. From 3 to 5 repeat VOP readings are taken in rapid succession (taking ~3 minutes) and averaged. The manually applied pressure via the force transducer's probe tip is less than generated when rubbing one's own eyes (max < 60 g).

The instantaneous predicted absolute intracranial pressure (ICP, mmHg) is derived from: 1) a linear transformation of the applied force (gms) into the resultant change in intraocular tension (IOT, mm Hg) by using a published nomogram [18] that incorporates a correction for the resting (baseline) intraocular pressure and 2) a second simple conversion of the IOT (at the VOP) into the predicted ICP based on the calibrated patient data. The previous reported ICP predictability by this method was r ~ 0.9 (Pearson's coefficient of linear correlation) [18].

### Clinical calibration and validation

#### Intra and inter-examiner accuracy

Reliability between serial measurements performed by the same examiner on a given patient and in the same environment is synonymous with 'repeatability' or 'measurement error' as reflected in the standard deviation of replicates. How accurate the instrument and technique are in the hands of another operator is a measure of 'agreement'. In previous work, the intra-examiner variance in VOP measurements was ± 9% in the intermediate range of venous occlusion pressures (10-25 gms). The inter-examiner variance in the means of replicates was ± 7% in control subjects [18]. In new studies involving normal subjects, we found the repeatability coefficient to be 3.9 gms (Additional file 2A), which corresponds to a 95% confidence limit (2 SD) of ± ~ 4 mm Hg (after nomogram conversion). The inter-observer agreement was shown to be slightly less with a coefficient of 4.7 grams, but is operationally acceptable given a less than 4 mm Hg difference between the mean ICPs estimated by different operators on a given subject (Additional file 2B). The pooled day-to-day variability among 3 additional subjects tested over 2-3 days was calculated at a similar ± 4.8 gms (95% CI, results not shown).

#### Prediction of actual ICP

It was established in prior work [17,18] that ODM calibrates linearly with actual ICP measurements obtained in critically ill patients using invasive cannulation and continuous monitoring. So that the field readings taken with the new device could be translated into absolute ICP (mm Hg) with confidence, we repeated the standardization in 12 hospitalized patients, most with hydrocephalus, requiring ventriculostomy or lumbar drainage (n = 16 encounters).

Ventriculostomies were assembled for continuous ICP monitoring with a calibrated pressure transducer set at the foramen of Monroe. The indications were for the management of hydrocephalus and intracranial hypertension due to hemorrhage, tumor or trauma (Table 1). Measurements were taken at 15° head elevation. Stable absolute mean ICPs were required for at least 1 hour before the non-invasive recordings were initiated. Where ventriculostomy provided the 'gold standard' ICP value, vODM were carried out in the 'open to drainage' position and clamped when possible, providing thus two points. The vODM procedure was carried out under institutional review board (IRB) approval following HIPAA guidelines and informed consent protocol. Subjects were excluded if they had carotid occlusion or severe stenosis, retinal disease, corneal opacification or acute glaucoma. Other grounds for exclusion were if the patient could not be positioned at 15° due to pressure support or if the subject could not maintain ocular fixation for any reason. While the presence of papilledema was not exclusionary, no subject had greater than grade 2. The VOP measurements (in gram units of load) were collected by one of 3 trained operators who were 'blinded' with respect to the medical details and actual ICP. An ICU nurse or assistant obscured the monitor from the operator's view and recorded the mean ICP and print out of the ICP waveform for placement in a sealed envelope with the neurosurgery study coordinator. At the end of the study, the seal was 'broken' with a collaborating neurosurgeon present.

**Table 1:** Patient Characteristics

Pt	Age/sex	Diagnosis	ICP method	MAP	mean IOP	ICP	mean VOP (sd)	NIHSS/GCS
1	71 M	NPH	Lumbar drain, intraoperative	98	15	11.7	17.4 (4.7)	0/15

2	53 M	R. temp ICH, craniotomy	Ventriculostomy.	97	10	7.0 (open) 22.5 (closed)	9.2 (1.0) 21.8 (2.8)	40×/5

3	61 M	Cerebell ICH	Ventriculostomy	108	4	11.0 (open) 40.6 (closed)	11.7 (2.4) 35.0 (3.3)	40×/3

4	61 F	Obstruct. HC TBI: IVH,SAH	VP shunt, intraoperative	89	15	8.8	11.8 (0.9)	1/14

5	83 F	NPH	VP shunt, intraoperative	102	21	13.2	23.8 (2.4)	0/15

6	46 M	Obstruct. HC congenital	VP shunt, intraoperative	102	14	25.0	31.5 (6.2)	0/15

7	53 M	R.temp-parietal ICH,IVH, craniotomy	Ventriculostomy	112	6	17.0 (open)	21.0 (4.5)	20/13

8	51 M	Communic. HC old TBI	Lumbar puncture	76	23	23.5	25.8 (3.9)	0/15

9	42 F	Vermis tumor, craniotomy	Ventriculostomy	105	7	10.0 (open)	10.9 (2.9)	NA/12

10	52 M	R. frontal contusion, TBI	ICP monitor	96	13	1.0	7.1 (1.6)	NA/6

11	22 M	Meningitis, acute HC	Ventriculostomy	110	10	24.0 (open)	20.6 (2.8)	42/4
						33.0 (closed)	26.9 (2.9)	
						9.0 (open)	12.2 (0.7)	

12	50 M	L. pariet-occip AVM: ICH,IVH	Ventriculostomy	73	14	11.5(closed)	13.4 (3.9)	2/14

Notes: All patients, except no. 10, evidenced hydrocephalus on brain imaging studies. Mean arterial, intraocular, intracranial and VOP pressures are in mm Hg. n = 16 observations, 8 O.D., 8 O.S.

Abbreviations: MAP: mean arterial pressure, IOP: intraocular pressure (resting), ICP: intracranial pressure, VOP: central retinal venous occlusion pressure, sd 1 standard deviation, NIHSS: Nat. Instit. Health Stroke Scale, GCS: Glasgow Coma Scale, NPH: normal pressure hydrocephalus, HC: hydrocephalus, AVM: arteriovenous malformation, VP: ventriculoperitoneal, TBI traumatic brain injury, IVH: intraventricular hemorrhage, SAH: subarachnoid hemorrhage, ICH: intracranial hemorrhage, O.D., O.S.: oculus dexter and sinster, NA not available

### High Altitude Measurements

#### vODM and subjects

On the southwestern approach to and South Col route on Mt. Everest (Nepal), we performed ODM measurements on 42 volunteers (a 'convenience set'), including multiple altitudes on 9 subjects. This research plan was part of a larger Brown University sponsored study in 2007 tracking cognitive and speech-motor control deficits at extreme altitude which was approved by the Nepal Health Research Council (NHRC). There were no exclusionary criteria as to age, sex, nationality or use of medications. All subjects were fluent in English and a similar disclosure and signed consent protocol was followed. The indigenous local population was not tested due to language barriers, their lifelong adaption to altitude and multiple ascent/decent profiles. At each climber-clinician encounter, a rapid succession of non-invasive vODM ICP readings (3-5) was obtained and an AMS/headache scale score was assessed by the operator (Lake Louise Consensus on Definition of Altitude Sickness, 1991. http://www.high-altitude-medicine.com). Thus, the investigator was not blinded to the AMS scoring. Tests were performed at sea level, in Kathmandu and at various stations up to and including upper base camp (17,500 ft 5400 m) and upper camp 2 (21,500 ft, 6600 m). They were made within 48 hours of arrival and on the ascent profile to a particular altitude.

**Figure 1:**
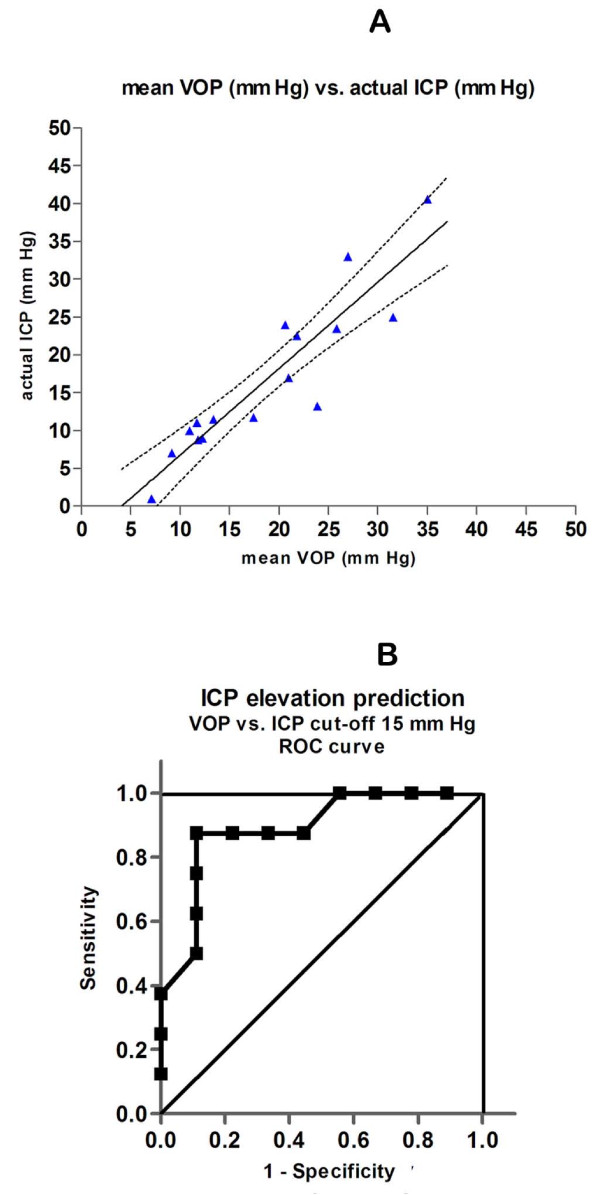
**Validation of vODM**. **A**. ODM measurements of the venous occlusion pressure (VOP, mmHg) conform linearly with actual ICP measurements made in critically ill patients with invasive canulation and continuous monitoring or taken in the OR at time of shunting or obtained through lumbar puncture. 12 patients with acute or decompensated hydrocephalus from various etiologies (intracranial hemorrhage n = 4, obstructive n = 2, 'NPH' = 2, communicating n = 2) and 1 each with acute trauma and midline tumor removal, underwent either ventriculostomy or lumbar puncture. They were measured in a total of 16 encounters by either of 3 operators who were blinded to the actual instantaneous ICP. The recorded ICPs ranged from 1.0 to 40.6 mm Hg. The individual points are the mean of 3-5 rapidly successive ODM readings of the VOP (mmHg) plotted against a single actual ICP reading (the fitted line is ICP = 1.07 VOP- 4.6; r = 0.85; (- - - -) denote 95% confidence limits). **B**. ROC curve for vODM data in A. Area under curve (AUC) is 0.89 (95% CI 0.73-1.05, p < .01).

The conversion from the VOP to estimated ICP requires a correction for the resting intraocular pressure [18]. Therefore, the rest IOP was obtained on all volunteers, before the intraocular tension (IOT) was mechanically increased. To reduce bias in making repeated intrasubject measurements, raw data was transcribed by a student assistant and the records not analyzed for ICP estimation until the conclusion of the study. The ICP of each individual at a given altitude station was estimated using first a published nomogram (Figure one in [18]) which converts the pressure applied to the eye (grams) to the resulting intraocular tension (mmHg). The IOT corresponding to the pressure at the point of CRV collapse is the VOP (mmHg). The ICP is then estimated from this VOP value using published correlation data (Figure three A in [18]) or in the case of this study, the calibration plot shown in Figure 1 (see also Figure four in [17]) (calculations not shown).

#### Psychometric Test

In a sub-aim of this study, a verbal-auditory word recall test was used to rapidly assess working memory (Additional file 3). The participant was read once only, a supraspan list of 21 simple familiar words in 30 seconds. Immediately thereafter, they were asked to recall all 7 words that conformed to a specific category. The specific category for each trial was presented to the subject before the list was read. He/she was instructed to ignore the extraneous words when responding. Four such lists were used. If the same subject was tested at more than one altitude, he/she was presented an alternate list. The four strategies were to recall words: beginning with the letter B, corresponding to the quality of being round, denoting a tool and categorized as an action or verb. At sea level, normal young adults achieved a mean of 6.0 ± 0.2 words irrespective of the particular list. The test is similar to the item category recall portion of the CVLT; the usual score is similar to that of other short word list tests (e.g. mean 5.6 [33]).

### Statistical Considerations

The VOPs from calibration vODM measurements performed on ICU patients were fitted to the response variable (absolute ICP) using standard linear regression by least squares. The VOP of climbers was correlated to altitude and AMS score in like fashion. A calculation for the number of observations required to power (1-β) the ICP vs. altitude Everest field study at .90 (α = .05) came to n = 42. It is based on the previously obtained Pearson correlation coefficient of 0.89, a p = .05 level of significance and a conservative inter-examiner and intra-subject variation of 9 mm Hg. A clinically important difference to detect was taken as 10 mm (effect size). For this study, Bland-Altman plots were used to evaluate repeatability and agreement [34-36] (Additional file 2A and 2B). vODM accuracy to discriminate normal from elevated ICP was assessed by constructing receiver operator characteristic (ROC) curves (GraphPad Prism Inc. statistical software).

## Results

The vODM device as depicted in Additional file 1 was calibrated using 12 hospitalized patients in the neuroICU or operating room and in whom ICP determination or monitoring was deemed medically necessary for such conditions as are noted in the Table 1. Most had hydrocephalus: 2 obstructive (decompensated congenital, subacute due to TBI), 2 idiopathic NPH, 2 communicating (decompensated old TBI, acute meningitis), and 4 with intracranial hemorrhage. In addition, 1 patient was post midline tumor resection and 1 had acute TBI with cerebral contusion. The conventional mean ICPs recorded from the transducers ranged from 1.0 to 40.6 mm Hg. The results summarized in Figure 1A were independently collected by three operators working in separate institutions, who were blinded to the simultaneous actual ICPs. The mean of 3-5 rapid, successive non-invasive ODM readings of the venous occlusion pressure (VOP) is highly correlated to the actual ICP (r = 0.85). This calibration confirms our earlier unblinded results [18] and are also very similar to the correlation published by another group using ODM, but on different instrumentation [17]. The ROC curve for this data set using ICP>15 mm Hg as the cutoff denoting elevated ICP, indicates a relative accuracy of 0.89 (area under curve; 95% CI is 0.73-1.05) to predict raised ICP (Figure 1B).

**Figure 2:**
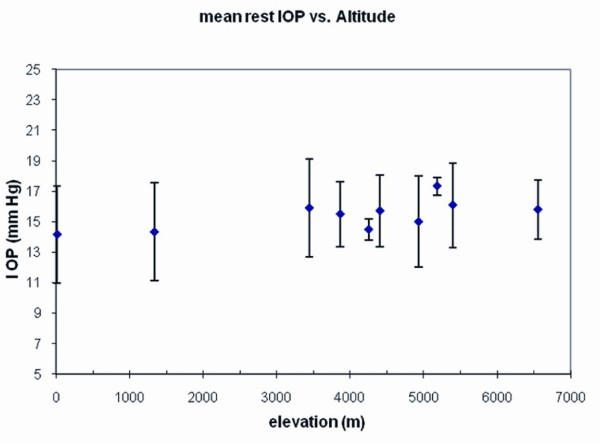
**Resting intraocular pressure**. Before introcular tension is raised by the ODM to determine the additional pressure required to observe the collapse of the central retinal vein, the baseline intraocular pressure (IOP) is taken from a tonometer reading. For every ODM measurement in the Mt. Everest cohort, these are plotted against elevation. There is no significant correlation of mean IOP with change in altitude. Values in mm Hg are ± 1 SD.

We next tested 42 trekkers and climbers on the ascent of Mt. Everest at various altitudes for a total of 54 VOP measurements. These were performed at elevations ranging from sea level to base camp (17,500 ft, 5400m) and at camp 2 (21,500 ft, 6553 m). First we found that the resting IOP did not change appreciably with altitude (Figure 2). This result is consistent with a very small reported change in mean IOP within 1-3 days after attaining 5200 m (from 11.4 to 12.4 mm Hg) [37]. Therefore, its overall effect on the CRV- and ICP-pressure correlation was minimal.

**Figure 3:**
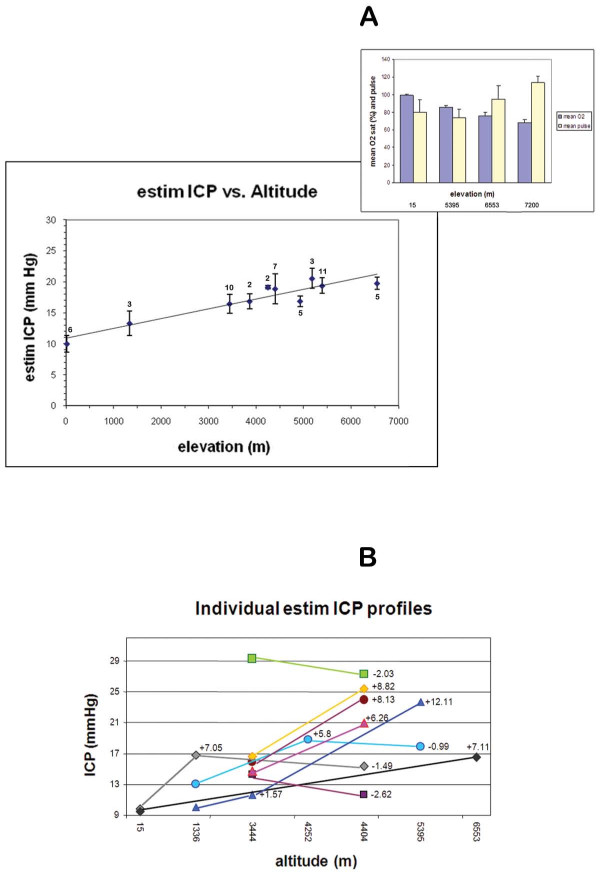
**Predicted ICP generally increases with elevation**. **A**. A total of 42 normal adult volunteer subjects were administered from 1 to 3 ODM measurements at varying elevations for a total of 54 encounters. Altitudes ranged from sea level (15 m) to upper basecamp on the Everest trek (5400 m) and upper Camp 2 (6600 m) on the mountain. From these measurements, the resting tonometry results of Figure 3. and published nomogram [18], the estimated ICP was calculated (mm Hg). Each point represents the mean of 3-5 readings from a given encounter, averaged again over the number of individuals (n) measured at the elevations shown below. Bars are ± 1 SE. By linear regression, r = 0.88. Mean ICP at sea level was 10.0 ± 3.4 (SD, n = 6), at Namche Bazaar (3445 m) was 16.5 ± 4.7 (n = 10, p < .005), at basecamp was 19.3 ± 4.1 (n = 11, p < .001) and at Camp 2 was 19.8 ± 2.1 (n = 5, p < .001). Resting oxygen saturations and pulse (± SD) were obtained at sea level (15 m, n = 6), r=0.87; basecamp (5400 m, n = 7), Camp 2 (6553 m, n = 7) and Camp 3 (7200 m, n = 5) indicating progressive hypoxia. **B**. Individual variations in mean calculated ICP are plotted where the same subject underwent repeat measurement at a higher elevation. N = 9 subjects for a total of 12 interval changes. The interval difference is denoted with + (increase) or - (decrease).

Next, we applied vODM to mechanically increase the intraocular tension to find the retinal venous occlusion pressure (VOP). The ICP at any given altitude and subject is estimated from the resting IOP and a nomogram converting the VOP into the corresponding induced IOP (mmHg) and a second linear transformation relating this to actual ICP (Figure 1). Compared to sea level measurements performed on young adults, in which the mean ICP was ~10 mm Hg, there was a clear trend for mean ICP to rise with elevation, reaching significance starting at 3445 m (Namche Bazaar)(p < .005). The mean estimated ICP at Camp 2 (6553 m) was ~20 mm Hg. A linear model as shown in Figure 3A, results in a calculated r = 0.88. Alternatively, there may be a leveling-off of ICP after 4300 m. Further studies involving higher elevations may be necessary to resolve this latter point. However, hypoxia is likely to be a major factor in this response and as expected, oxygen desaturation is significant at basecamp and progressively worsens with ascent to Camps 2 and then 3 in a subset of climbers (Figure 3A, **inset**). Among individual subjects that underwent repeat testing at different elevations, the trend also reflected an increase at the higher elevation (Figure 3B).

**Figure 4:**
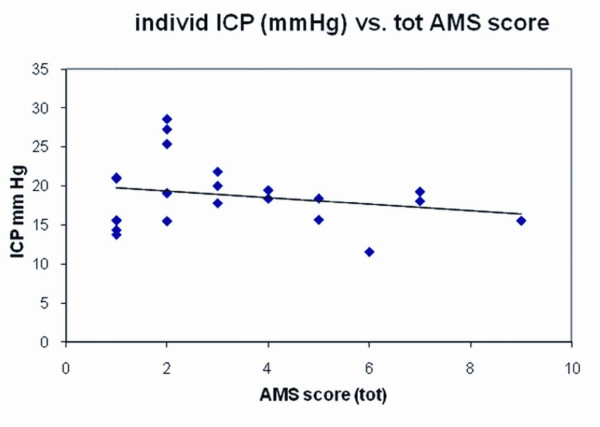
**AMS symptom score vs. estimated ICP**. 22 volunteers had AMS symptoms ranging from 1 (e.g. mild headache) to 9 (e.g. incapacitating headache with nausea, dizziness and sleep disturbance). AMS plotted against ICP shows a relative lack of correlation (r = 0.05).

Almost one half of all volunteers had one or more symptoms of acute mountain sickness. Corresponding to mild severity, these ranged in symptom score from 1-9 (Lake Louise criteria, see methods). When plotted against the predicted ICP at the time of assessment, there was little evidence for correlation (Figure 4; r = .05).

## Discussion

These results support the feasibility that absolute ICP can be estimated and that non-invasive vODM has practical utility by showing changes that correlate predictably with a relevant physiologic variable (hypoxia). To our knowledge, these preliminary studies are the first to use ophthalmodynamometry in the high altitude setting. We improved upon our earlier calibration and validation studies of vODM in using a new set of ICU patients under a 'blinded' protocol. This approach to non-invasive ICP measurement is relatively direct because the level of induced intraocular pressure where the retinal vein is seen to collapse, is an unequivocal dynamic endpoint. There is also a direct relationship to the pressure in the intracranial compartment into which the venous retinal blood flow has to return. These properties make the prediction of ICP in absolute mm Hg possible.

Although there is considerable individual variation, the general increase in intracranial pressure with altitude probably represents a universal physiologic response to hypoxia. This is in agreement with previous MRI findings of cerebral swelling [38,39]. The mechanisms behind the cerebral intravascular expansion and/or vasogenic edema are not known. Hypoxia is suspected to stimulate cerebral blood flow (and to increase brain blood volume) but this literature is contentious. Simulated altitude studies show CBF increases acutely after attaining elevations up to 4559 m [40-44]. Others found no change [45-47]. Variances in vascular reactivity (e.g. controlling for exercise) and cerebral blood vessel autoregulation (e.g. controlling for systemic blood pressure) may underlie some of these differences. BBB disruption due to hypoxemia and reactive oxyradical stress [21,23] has been implicated in edema formation. However, no changes in vascular permeability or cerebral edema accompanied rising markers of oxidative stress in a hypoxic chamber - AMS study [26]. Moreover, one hypoxia-induced angiogenic factor that promotes capillary leakage, VEGF [48-51], has been excluded of association with either hypoxia or AMS [26]. Exhaled levels of the vasodilator nitric oxide (pNO) decrease with altitude but also fail to correlate with AMS symptoms [52].

Importantly, no correlation with AMS was found in most of the above studies, with only 2 exceptions [41,53]. Similar to the MRI studies on brain volume, we found no relationship of the increase in ICP to the incidence or severity of mild-mod AMS symptoms. Alternatively, the etiology of headaches could reflect vasoactive instability but does not exclude a lesser contribution from cerebral swelling and rise in ICP. Indeed, a less compliant ventricular CSF system is proposed to predispose AMS [32]. Chemical mediators, such as NO and arachadonic acid metabolites, activate trigeminovascular system pain fibers which travel in proximity to meningeal blood vessels [54]. In combination with mechanical stretch from local edema or intracranial hypertension (ICH), this migraine-like hypothesis may explain individual responsivity to anti-migraine therapies for AMS such as NSAIDS [55], sumatriptan [56-58] and analgesics (personal observation).

Another disabling symptom of high altitude, cognitive impairment, particularly working memory and executive control, is reported in human [59] and rodent [60-62] studies. Various hypoxic conditions (e.g. sleep apnea, exhaustive exercise) are associated with reductions in prefrontal oxygenation or activation [63-65]. Working memory impairment at altitude is consistent with frontal-subcortical vulnerability to hypoxia [66,67]. There is no data on whether ICP has any independent effect on frontal-based functions. We found a general decline in a working memory test at the two highest elevations (Additional file 3A). Although the result is expected, there was great variability among subjects. Similarly, we found only weak negative correlation between test performance and estimated ICPs (r = 0.64, Additional file 3B).

Our study confirms one aspect of an older [68] and more recent non-invasive study on Mt. Everest that used a very different ocular technique [69]. Optic nerve sheath diameter (ONSD) assessment by ultrasound showed increases in optic nerve diameter with altitude up to 6400 m (21,000 ft). Since nerve sheath diameter is an indirect indicator of ICP [16], the authors conclude, as we do, that brain pressure naturally rises. However, they and recently another group [70] oppositely report a positive correlation with mean AMS scores, concluding that ICP increases are a major factor in AMS pathophysiology.

All non-invasive techniques have limitations. ONSD values are relative and not calibrated to actual ICP. Moreover, intrathecal infusion tests show that ONSD responses are linear only between 22 and 30 mm Hg [16] and in ICU patients, the scatter in ONSD vs. ICP yielded a correlation coefficient of 0.59. A cut-off of 5 mm ONSD was determined to provide a relative assessment of ICPs > 20 cm H2O [71]. Regarding ODM measurements, changes in retinal blood flow at altitude could in theory confound the interpretation of VOP measurements. Indirect estimates at 17,500 feet [72] or more direct flow measurements but after descent [73], suggest such increases can occur. Thus, increasing VOPs at higher altitude may reflect a combination of ICP and increasing blood volume in the retinal venous circuit. How retinal blood flow and volume affect ODM measurements is an important relationship to establish in future research. Another variable possibly affecting both techniques is that local interstitial edema, as may involve multiple body organs at high altitude, could affect either ONSD or ocular vascular pressure readings independent of brain pressure. Assessment of this contribution under field conditions posses another challenge.

Several limitations of our pilot altitude study are acknowledged. First, the readings were not 'blinded' since they were collected while the examiner was aware of the altitude and AMS score. Our study, unlike the ONSD report, was not a longitudinal one by design. It was carried out on a variable dataset consisting of a small number of volunteers (≤10) at most altitudes. Together with the primitive or extreme conditions, a low number of measurements is understandable. Had an even larger proportion of our subjects been repeatedly measured at various altitudes, a greater power of statistical association may have been obtained. These issues and the differences in the role of ICP as a factor in AMS aggravation concluded by the ODM and ONSD data could be resolved through a larger trial on a single cohort. Other features to build into subsequent studies are to manipulate ICP (e.g. increase PaCO_2_ or 0_2_) [74], potentially treat AMS and make additional measurements on the descent phase. Nevertheless, our study affords a practical approach to AMS evaluation by proof-of-principle that a symptomatic individual can be spot evaluated for actual ICP.

## Conclusion

vODM is a safe and validated method to predict ICP as shown in this new blinded study of hospitalized patients with acute or decompensated hydrocephalus of varying etiologies. Validation for field use was explored in a small sample of normal volunteers under hypobaric hypoxic conditions. In this pilot study of trekkers and veteran climbers on Mt Everest, there is evidence for a significant general increase in intracranial pressure with altitude. However, this moderate increase did not appear to be causal in the occurrence of mild to moderate acute mountain sickness.

## Abbreviations

ICP: intracranial pressure; vODM: venous ophthalmodynamometry (-meter); CRV: central retinal vein; ON: optic nerve; AMS: acute mountain sickness; HACE: high altitude cerebral edema; WM: working memory; IOP: intraocular pressure; ICH: intracranial hypertension; VOP: venous occlusion pressure; CSF: cerebral spinal fluid; PFC: prefrontal cortex; TBI: traumatic brain injury.

## Competing interests

This study was partially funded by a grant through the Massachusetts Technology Transfer Council (MTTC) to HWQ. No author has a business relationship with or membership status on the council. A US patent on vODM technology is held by Caritas St. Elizabeth's Medical Center (CSEMC). HWQ is a non-paid consultant to Third Eye Diagnostics Inc., Pennsylvania USA. Neither MTTC nor Third Eye Inc. participated in or reviewed any aspect of this manuscript and have no financial interest in this work. The authors have not received or anticipate receiving any fees from this work and declare no competing financial interests.

## Authors' contributions

HWQ is principle investigator and author of this manuscript. PL participated in data analysis and manuscript editing. SA and SM engineered the vODM device. MB and CvH assisted in data collection, interpretation and manuscript editing. All authors read and approved the final manuscript.

## Pre-publication history

The pre-publication history for this paper can be accessed here:

http://www.biomedcentral.com/1471-2377/10/106/prepub

## Supplementary Material

Additional file 1 **Schematic and actual venous ophthalmodynamometer (ODM)**. The finger button-activated freeze display and reset switch is shown tethered to the device on the far right. The force transducer (DVRT- eye pressure sensor) is shown over the circuitry board, center right, with attached scleral footplate and cable. Two 14.8 V Lithium cells are seen to the left, below a transparent protective seal from Lucite. The liquid crystal display readout is calibrated in grams. View of Western Cwm, Camp 2 in the foreground, Lhotse center, shoulders of Everest (left) and Nuptse (right). Click here for file

Additional file 2 **Validation studies**. **A**. Repeatabilty is the accuracy with which an operator obtains the same venous occlusion pressure (gms) while making multiple measurements on the same subject. Results from 2 subjects are shown. 10 single measurements of subject A over 3 days netted a mean of 7.1 gms. The average of 12 back-to-back measurements on another subject B was 10.1 gms. The differences from the means are plotted. The 2SD levels indicate the repeatability coefficient of 3.9 gms (95% confidence limits). **B**. Reproducibility is the accuracy with which 2 operators agree on VOP measurements. Interobserver differences in paired measurements from the pooled mean are plotted for 3 new subjects, A, B, and C. Operator no.1 was compared with no.2 for the first two subjects and operator no.1 compared to a third (no. 3) for subject C. The respective means obtained by each operator (force, grams), blinded to the results of the other, are given below and indicate good agreement (coefficient = 4.7 gms). Bland-Altman plots. Click here for file

Additional file 3 **Working memory and altitude**. **A**. In a preliminary assessment of this executive cognitive function at increasing altitude, all subjects were administered a 30 sec word recall test involving the immediate recall of a best of 7 in-category words from a read list of 21. The test always followed the ODM exam. Performance varied considerably between individuals but trended downward at basecamp and higher elevation (±1 SD). **B**. Mean word recall at increasing intervals (2 mmHg) of estimated ICP also show a downward trend, but not reaching significance. Click here for file
