# Supplementary figures and images for: Ophthalmodynamometry for ICP prediction and pilot test on Mt. Everest
Source: BMC Neurol. 2010 Nov 1;10:106. doi: 10.1186/1471-2377-10-106 (PMC2987855; doi:10.1186/1471-2377-10-106)

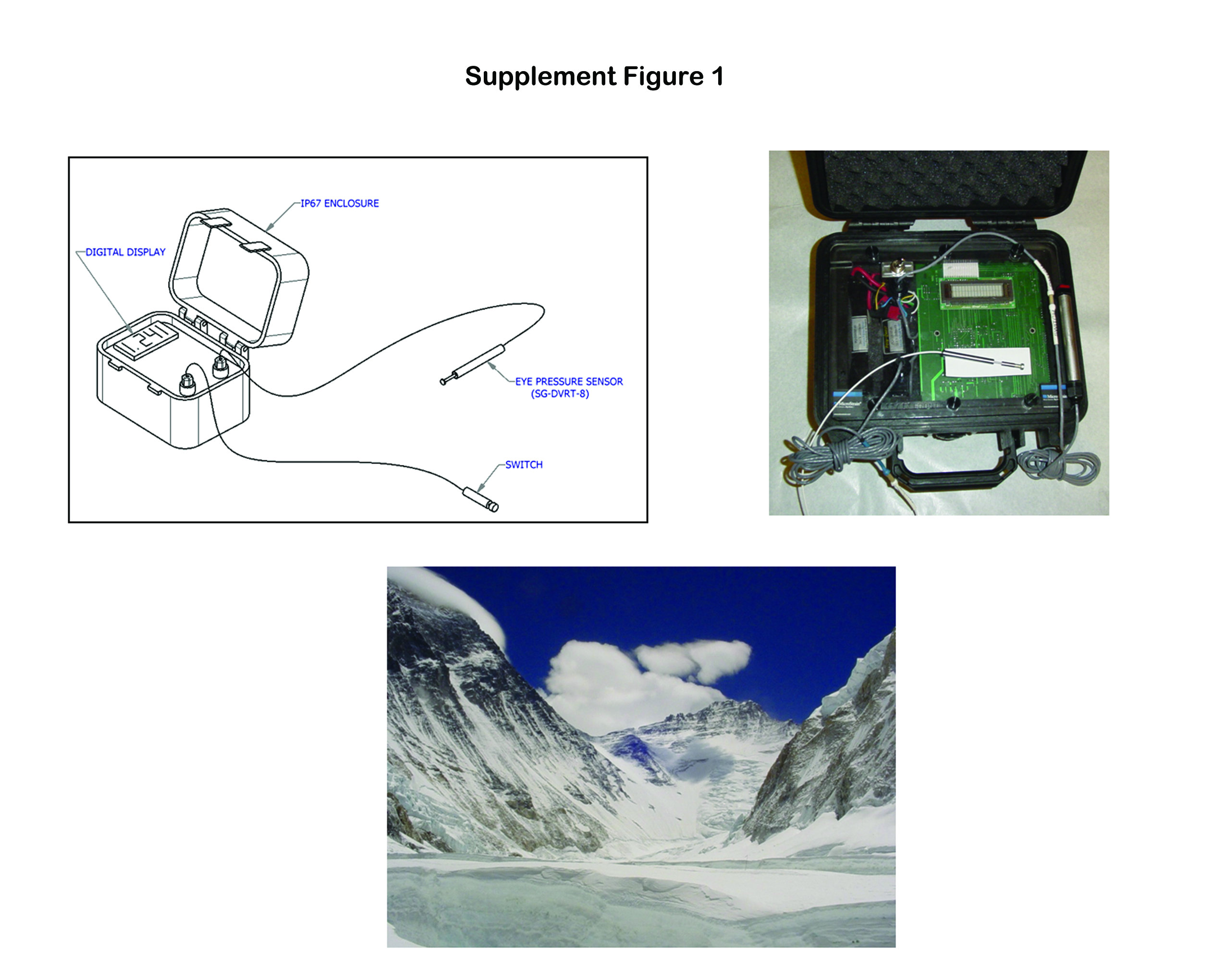

Supplement: Additional file 1 — Schematic and actual venous ophthalmodynamometer (ODM). The finger button-activated freeze display and reset switch is shown tethered to the device on the far right. The force transducer (DVRT- eye pressure sensor) is shown over the circuitry board, center right, with attached scleral footplate and cable. Two 14.8 V Lithium cells are seen to the left, below a transparent protective seal from Lucite. The liquid crystal display readout is calibrated in grams. View of Western Cwm, Camp 2 in the foreground, Lhotse center, shoulders of Everest (left) and Nuptse (right). [file 1471-2377-10-106-S1.TIFF]

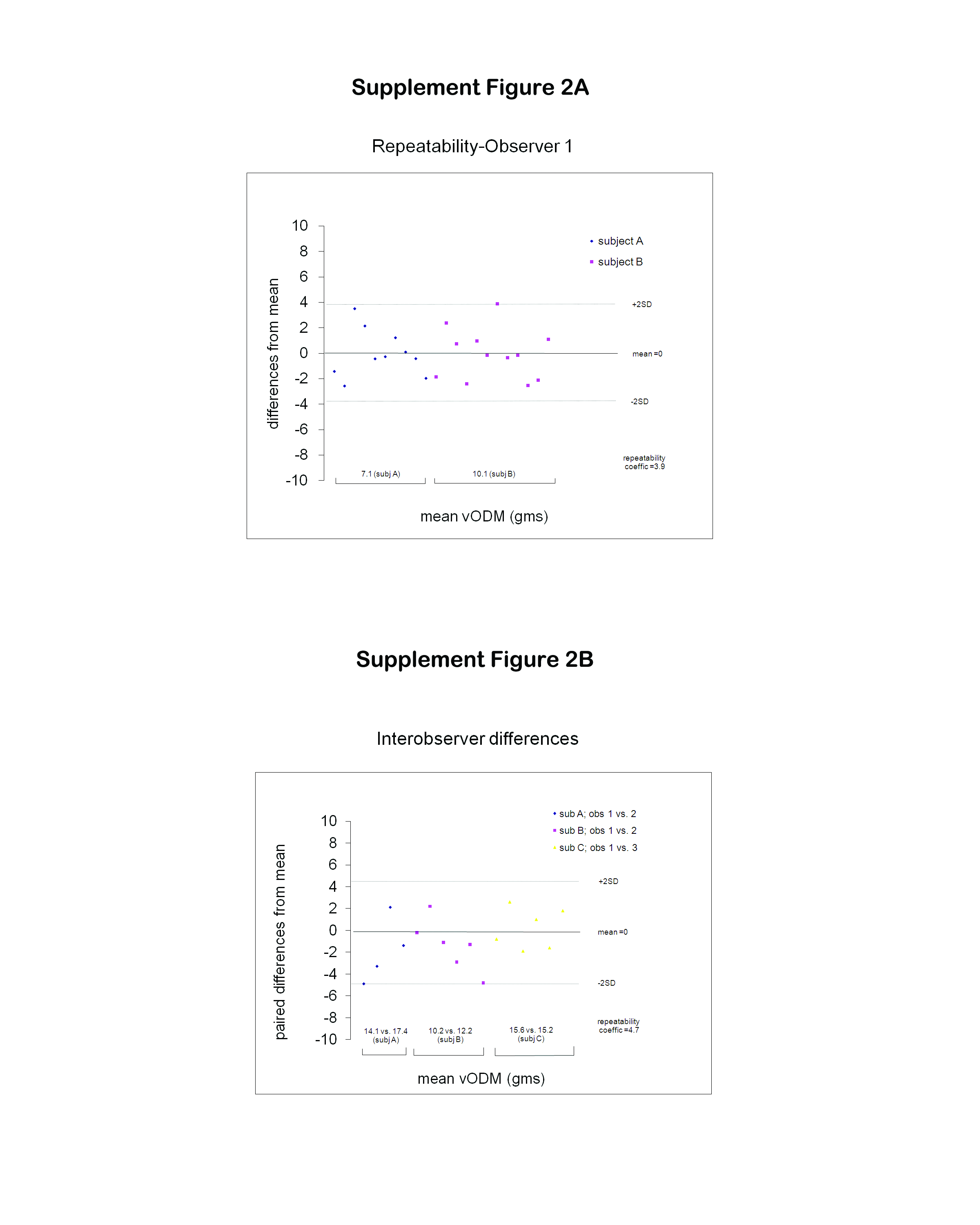

Supplement: Additional file 2 — Validation studies. A. Repeatabilty is the accuracy with which an operator obtains the same venous occlusion pressure (gms) while making multiple measurements on the same subject. Results from 2 subjects are shown. 10 single measurements of subject A over 3 days netted a mean of 7.1 gms. The average of 12 back-to-back measurements on another subject B was 10.1 gms. The differences from the means are plotted. The 2SD levels indicate the repeatability coefficient of 3.9 gms (95% confidence limits). B. Reproducibility is the accuracy with which 2 operators agree on VOP measurements. Interobserver differences in paired measurements from the pooled mean are plotted for 3 new subjects, A, B, and C. Operator no.1 was compared with no.2 for the first two subjects and operator no.1 compared to a third (no. 3) for subject C. The respective means obtained by each operator (force, grams), blinded to the results of the other, are given below and indicate good agreement (coefficient = 4.7 gms). Bland-Altman plots. [file 1471-2377-10-106-S2.TIFF]

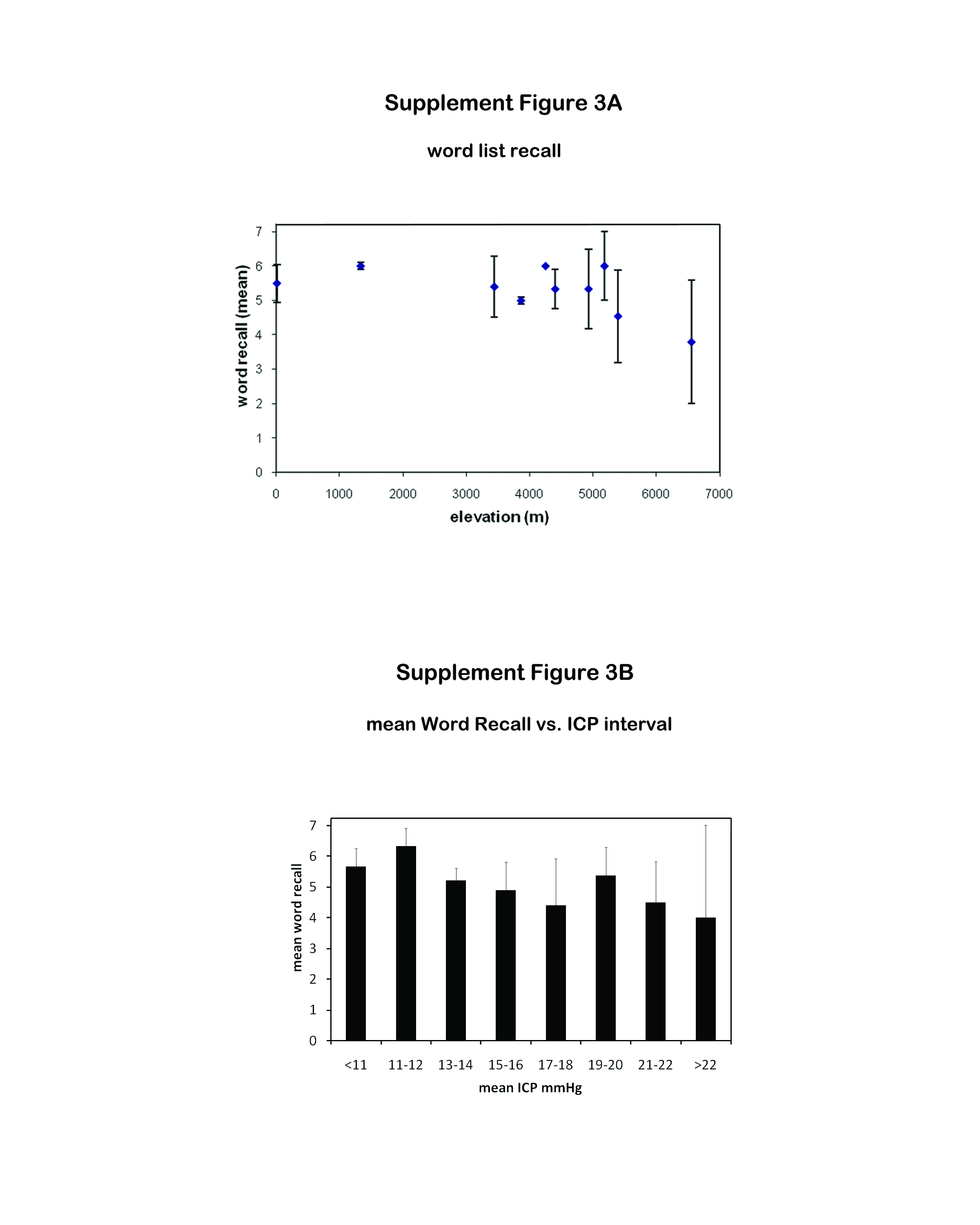

Supplement: Additional file 3 — Working memory and altitude. A. In a preliminary assessment of this executive cognitive function at increasing altitude, all subjects were administered a 30 sec word recall test involving the immediate recall of a best of 7 in-category words from a read list of 21. The test always followed the ODM exam. Performance varied considerably between individuals but trended downward at basecamp and higher elevation (±1 SD). B. Mean word recall at increasing intervals (2 mmHg) of estimated ICP also show a downward trend, but not reaching significance. [file 1471-2377-10-106-S3.TIFF]
